# 1-(2-Hydr­oxy-5-methyl­phen­yl)ethanone [(1*H*-indol-3-­yl)acet­yl]hydrazone

**DOI:** 10.1107/S1600536808011124

**Published:** 2008-04-26

**Authors:** Hapipah M. Ali, Kadir Zuraini, Basirun Wan Jeffrey, Mohd. Razali Rizal, Seik Weng Ng

**Affiliations:** a Department of Chemistry, University of Malaya, 50603 Kuala Lumpur, Malaysia

## Abstract

The indolyl –NH group of the title Schiff base, C_19_H_19_N_3_O_2_, forms a hydrogen bond to the –OH group of an inversion-related mol­ecule, resulting in a hydrogen-bonded dimer; adjacent dimers are further linked through an inter­dimer N—H⋯O hydrogen bond involving the –C(=O)–NH–N=fragment to form a linear ribbon that runs along the *a* axis.

## Related literature

For a related compound that co-crystallizes with 3-indolylacetyl­hydrazine, see: Ali *et al.* (2007 ▶).
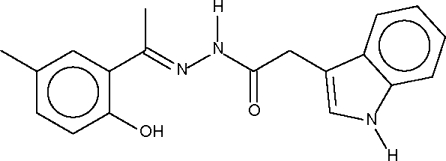


## Experimental

### 

#### Crystal data

C_19_H_19_N_3_O_2_
*M* *_r_* = 321.37
Triclinic, 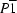

*a* = 4.6812 (9) Å
*b* = 12.419 (3) Å
*c* = 14.202 (3) Å
α = 109.919 (3)°
β = 91.710 (3)°
γ = 90.751 (3)°
*V* = 775.7 (3) Å^3^
*Z* = 2
Mo *K*α radiation
μ = 0.09 mm^−1^
*T* = 100 (2) K
0.40 × 0.13 × 0.05 mm

#### Data collection

Bruker SMART APEX diffractometer
Absorption correction: none
4854 measured reflections
3490 independent reflections
1905 reflections with *I* > 2σ(*I*)
*R*_int_ = 0.051

#### Refinement

*R*[*F* ^2^ > 2σ(*F* ^2^)] = 0.060
*wR*(*F* ^2^) = 0.196
*S* = 1.01
3490 reflections
231 parameters
3 restraints
H atoms treated by a mixture of independent and constrained refinement
Δρ_max_ = 0.43 e Å^−3^
Δρ_min_ = −0.46 e Å^−3^

### 

Data collection: *APEX2* (Bruker, 2007 ▶); cell refinement: *SAINT* (Bruker, 2007 ▶); data reduction: *SAINT*; program(s) used to solve structure: *SHELXS97* (Sheldrick, 2008 ▶); program(s) used to refine structure: *SHELXL97* (Sheldrick, 2008 ▶); molecular graphics: *X-SEED* (Barbour, 2001 ▶); software used to prepare material for publication: *publCIF* (Westrip, 2008 ▶).

## Supplementary Material

Crystal structure: contains datablocks global, I. DOI: 10.1107/S1600536808011124/bt2687sup1.cif

Structure factors: contains datablocks I. DOI: 10.1107/S1600536808011124/bt2687Isup2.hkl

Additional supplementary materials:  crystallographic information; 3D view; checkCIF report

## Figures and Tables

**Table 1:** Hydrogen-bond geometry (Å, °)

*D*—H⋯*A*	*D*—H	H⋯*A*	*D*⋯*A*	*D*—H⋯*A*
O1—H1o⋯N1	0.85 (3)	1.75 (2)	2.540 (3)	153 (4)
N2—H2n⋯O2^i^	0.85 (3)	2.05 (3)	2.884 (3)	166 (4)
N3—H3n⋯O1^ii^	0.85 (3)	2.08 (3)	2.913 (3)	166 (3)

Symmetry codes: (i) 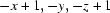; (ii) 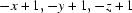.

**Fig. 1.**
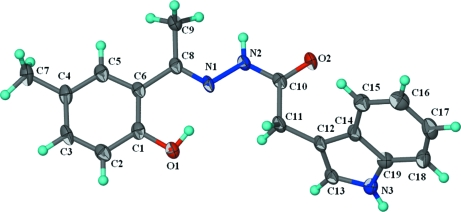
Thermal ellipsoid plot of C19H19N3O2; ellipsoids are drawn at the 70% probability level, and H atoms as spheres of arbitrary radii.

**Fig. 2.**
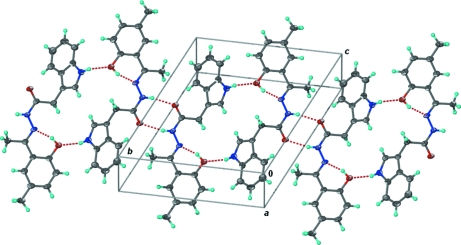
Hydrogen-bonded chain structure. Dashed lines denote H atoms.

## supplementary crystallographic information

### Comment

The Schiff base that is derived by condensing 2,4-dihydroxyacetophenone with indole-3-acetylhydrazine crystallizes as a co-crystal with unchanged indole-3-acetylhydrazine (Ali *et al.*, 2007). The reason for the formation of the co-crystal appears to be related to the ease of hydrogen bond formation as the parent ketone itself has two possible donor sites.

In the similar synthesis but with 2-hydroxy-5-methylacetophenone, only the pure Schiff base is obtained (Scheme I, Fig. 1). The indolyl –NH unit forms a hydrogen bond to the –OH unit of an inversion-related molecule to furnish a hydrogen-bonded dimer; adjacent dimers are further linked through an inter-dimer N–H···O hydrogen involving the –C(=O)–NH–N= fragment to form a linear ribbon chain that runs along the shortest axis of the triclinic unit cell (Fig. 2). The hydroxy group itself engages in intramolecular hydrogen bonding.

### Experimental

Indole-3-acetylhydrazine (0.55 g, 4 mmol) and 2-hydroxy-5-methylacetophenone (0.52 g, 4 mmol) were dissolved in ethanol (100 ml). The reactants were heated under reflux for 1 h. The solvent was removed to give the tSchiff base, which was purified by recrystallization from ethanol.

### Refinement

Carbon-bound H-atoms were placed in calculated positions (C—H 0.99 to 0.99 Å) and were included in the refinement in the riding model approximation, with *U*(H) set to 1.2 to 1.5*U*(C).

The amino and hydroxy H-atoms were located in a difference Fourier map, and were refined with a distance restraint of N–H/O–H 0.85±0.01 Å; their displacement parameters were freely refined.

### Crystal data

**Table:**

C_19_H_19_N_3_O_2_	*Z* = 2
*M**_r_* = 321.37	*F*_000_ = 340
Triclinic, *P*1	*D*_x_ = 1.376 Mg m^−^^3^
Hall symbol: -P 1	Mo *K*α radiation λ = 0.71073 Å
*a* = 4.6812 (9) Å	Cell parameters from 985 reflections
*b* = 12.419 (3) Å	θ = 3.0–28.3º
*c* = 14.202 (3) Å	µ = 0.09 mm^−^^1^
α = 109.919 (3)º	*T* = 100 (2) K
β = 91.710 (3)º	Plate, pale yellow
γ = 90.751 (3)º	0.40 × 0.13 × 0.05 mm
*V* = 775.7 (3) Å^3^	

### Data collection

**Table:**

Bruker SMART APEXII diffractometer	1905 reflections with *I* > 2σ(*I*)
Radiation source: fine-focus sealed tube	*R*_int_ = 0.052
Monochromator: graphite	θ_max_ = 27.5º
*T* = 100(2) K	θ_min_ = 1.5º
ω scans	*h* = −6→3
Absorption correction: none	*k* = −15→16
4854 measured reflections	*l* = −17→18
3490 independent reflections	

### Refinement

**Table:**

Refinement on *F*^2^	Secondary atom site location: difference Fourier map
Least-squares matrix: full	Hydrogen site location: inferred from neighbouring sites
*R*[*F*^2^ > 2σ(*F*^2^)] = 0.060	H atoms treated by a mixture of independent and constrained refinement
*wR*(*F*^2^) = 0.196	*w* = 1/[σ^2^(*F*_o_^2^) + (0.0894*P*)^2^] where *P* = (*F*_o_^2^ + 2*F*_c_^2^)/3
*S* = 1.01	(Δ/σ)_max_ = 0.001
3490 reflections	Δρ_max_ = 0.43 e Å^−^^3^
231 parameters	Δρ_min_ = −0.46 e Å^−^^3^
3 restraints	Extinction correction: none
Primary atom site location: structure-invariant direct methods	

### Special details

**Table:**

Geometry. All e.s.d.'s (except the e.s.d. in the dihedral angle between two l.s. planes) are estimated using the full covariance matrix. The cell e.s.d.'s are taken into account individually in the estimation of e.s.d.'s in distances, angles and torsion angles; correlations between e.s.d.'s in cell parameters are only used when they are defined by crystal symmetry. An approximate (isotropic) treatment of cell e.s.d.'s is used for estimating e.s.d.'s involving l.s. planes.

### Fractional atomic coordinates and isotropic or equivalent isotropic displacement parameters (Å^2^)

**Table:**

	*x*	*y*	*z*	*U*_iso_*/*U*_eq_	
O1	−0.1765 (5)	0.39859 (17)	0.64793 (15)	0.0232 (5)	
H1O	−0.097 (9)	0.335 (2)	0.617 (3)	0.072 (15)*	
O2	0.5909 (4)	0.08863 (17)	0.43759 (15)	0.0259 (5)	
N1	0.0800 (5)	0.21073 (19)	0.61339 (17)	0.0198 (5)	
N2	0.2703 (5)	0.1345 (2)	0.55630 (18)	0.0205 (6)	
H2N	0.287 (9)	0.0706 (17)	0.565 (3)	0.055 (12)*	
N3	0.8788 (6)	0.3894 (2)	0.33999 (18)	0.0224 (6)	
H3N	0.989 (6)	0.4440 (19)	0.339 (2)	0.021 (8)*	
C1	−0.2982 (6)	0.3739 (2)	0.7244 (2)	0.0201 (6)	
C2	−0.4852 (6)	0.4525 (3)	0.7818 (2)	0.0241 (7)	
H2	−0.5215	0.5206	0.7676	0.029*	
C3	−0.6195 (6)	0.4327 (3)	0.8597 (2)	0.0251 (7)	
H3	−0.7480	0.4874	0.8985	0.030*	
C4	−0.5699 (6)	0.3340 (3)	0.8822 (2)	0.0243 (7)	
C5	−0.3819 (6)	0.2562 (3)	0.8235 (2)	0.0226 (7)	
H5	−0.3487	0.1879	0.8377	0.027*	
C6	−0.2391 (6)	0.2731 (2)	0.7447 (2)	0.0184 (6)	
C7	−0.7165 (7)	0.3105 (3)	0.9666 (2)	0.0302 (8)	
H7A	−0.5727	0.2956	1.0119	0.045*	
H7B	−0.8452	0.2434	0.9390	0.045*	
H7C	−0.8269	0.3772	1.0037	0.045*	
C8	−0.0378 (6)	0.1881 (2)	0.6856 (2)	0.0196 (6)	
C9	0.0212 (7)	0.0829 (2)	0.7102 (2)	0.0282 (7)	
H9A	0.2248	0.0659	0.7027	0.042*	
H9B	−0.0926	0.0183	0.6646	0.042*	
H9C	−0.0299	0.0952	0.7794	0.042*	
C10	0.4156 (6)	0.1572 (2)	0.4841 (2)	0.0199 (6)	
C11	0.3479 (6)	0.2641 (2)	0.4612 (2)	0.0216 (6)	
H11A	0.1536	0.2549	0.4296	0.026*	
H11B	0.3454	0.3294	0.5251	0.026*	
C12	0.5528 (6)	0.2925 (2)	0.3940 (2)	0.0201 (6)	
C13	0.7169 (6)	0.3905 (2)	0.4185 (2)	0.0216 (6)	
H13	0.7181	0.4508	0.4813	0.026*	
C14	0.6163 (6)	0.2267 (2)	0.2925 (2)	0.0207 (6)	
C15	0.5219 (7)	0.1205 (3)	0.2253 (2)	0.0255 (7)	
H15	0.3838	0.0759	0.2444	0.031*	
C16	0.6313 (7)	0.0813 (3)	0.1311 (2)	0.0306 (8)	
H16	0.5677	0.0090	0.0851	0.037*	
C17	0.8355 (7)	0.1464 (3)	0.1020 (2)	0.0302 (8)	
H17	0.9079	0.1174	0.0365	0.036*	
C18	0.9324 (7)	0.2513 (3)	0.1664 (2)	0.0274 (7)	
H18	1.0698	0.2956	0.1467	0.033*	
C19	0.8215 (6)	0.2900 (3)	0.2617 (2)	0.0231 (7)	

### Atomic displacement parameters (Å^2^)

**Table:**

	*U*^11^	*U*^22^	*U*^33^	*U*^12^	*U*^13^	*U*^23^
O1	0.0203 (11)	0.0229 (11)	0.0256 (11)	0.0048 (9)	0.0064 (9)	0.0065 (9)
O2	0.0220 (12)	0.0258 (11)	0.0294 (11)	0.0088 (9)	0.0113 (9)	0.0077 (9)
N1	0.0119 (12)	0.0211 (12)	0.0228 (12)	0.0040 (10)	0.0046 (10)	0.0024 (10)
N2	0.0178 (13)	0.0187 (12)	0.0235 (12)	0.0040 (11)	0.0058 (10)	0.0048 (11)
N3	0.0184 (14)	0.0229 (13)	0.0264 (13)	0.0008 (11)	0.0045 (11)	0.0087 (11)
C1	0.0122 (14)	0.0250 (15)	0.0211 (14)	0.0001 (12)	−0.0006 (12)	0.0056 (12)
C2	0.0172 (16)	0.0236 (15)	0.0281 (16)	0.0032 (13)	−0.0008 (13)	0.0043 (13)
C3	0.0157 (15)	0.0297 (16)	0.0248 (16)	0.0078 (13)	0.0046 (13)	0.0022 (13)
C4	0.0135 (15)	0.0329 (17)	0.0237 (15)	0.0019 (13)	0.0026 (12)	0.0060 (13)
C5	0.0149 (15)	0.0263 (15)	0.0250 (15)	0.0020 (12)	0.0009 (12)	0.0064 (13)
C6	0.0118 (14)	0.0206 (14)	0.0202 (14)	0.0012 (12)	−0.0005 (11)	0.0036 (12)
C7	0.0214 (17)	0.0393 (19)	0.0274 (16)	0.0050 (15)	0.0085 (14)	0.0076 (14)
C8	0.0136 (15)	0.0210 (14)	0.0213 (14)	0.0005 (12)	0.0014 (12)	0.0032 (12)
C9	0.0292 (18)	0.0226 (15)	0.0329 (17)	0.0065 (14)	0.0135 (14)	0.0085 (14)
C10	0.0151 (14)	0.0196 (14)	0.0215 (14)	0.0026 (12)	0.0002 (12)	0.0023 (12)
C11	0.0171 (15)	0.0228 (14)	0.0244 (15)	0.0069 (12)	0.0046 (12)	0.0068 (12)
C12	0.0127 (14)	0.0238 (15)	0.0248 (15)	0.0078 (12)	0.0039 (12)	0.0091 (12)
C13	0.0173 (15)	0.0225 (15)	0.0252 (15)	0.0074 (12)	0.0045 (12)	0.0079 (12)
C14	0.0122 (14)	0.0263 (15)	0.0247 (15)	0.0061 (12)	0.0023 (12)	0.0098 (12)
C15	0.0224 (16)	0.0256 (15)	0.0267 (16)	0.0013 (13)	0.0012 (13)	0.0065 (13)
C16	0.0322 (19)	0.0290 (17)	0.0283 (17)	0.0021 (15)	−0.0023 (15)	0.0069 (14)
C17	0.0321 (19)	0.0381 (18)	0.0192 (15)	0.0105 (15)	0.0048 (14)	0.0074 (14)
C18	0.0219 (17)	0.0321 (17)	0.0300 (16)	0.0034 (14)	0.0078 (14)	0.0123 (14)
C19	0.0159 (15)	0.0263 (15)	0.0275 (16)	0.0064 (12)	0.0021 (13)	0.0096 (13)

### Geometric parameters (Å, °)

**Table:**

O1—C1	1.364 (3)	C7—H7C	0.9800
O1—H1O	0.85 (3)	C8—C9	1.491 (4)
O2—C10	1.226 (3)	C9—H9A	0.9800
N1—C8	1.288 (3)	C9—H9B	0.9800
N1—N2	1.375 (3)	C9—H9C	0.9800
N2—C10	1.352 (4)	C10—C11	1.505 (4)
N2—H2N	0.85 (3)	C11—C12	1.494 (4)
N3—C13	1.363 (4)	C11—H11A	0.9900
N3—C19	1.367 (4)	C11—H11B	0.9900
N3—H3N	0.85 (3)	C12—C13	1.365 (4)
C1—C2	1.381 (4)	C12—C14	1.436 (4)
C1—C6	1.406 (4)	C13—H13	0.9500
C2—C3	1.379 (4)	C14—C15	1.396 (4)
C2—H2	0.9500	C14—C19	1.404 (4)
C3—C4	1.389 (4)	C15—C16	1.376 (4)
C3—H3	0.9500	C15—H15	0.9500
C4—C5	1.386 (4)	C16—C17	1.404 (5)
C4—C7	1.508 (4)	C16—H16	0.9500
C5—C6	1.394 (4)	C17—C18	1.374 (4)
C5—H5	0.9500	C17—H17	0.9500
C6—C8	1.475 (4)	C18—C19	1.392 (4)
C7—H7A	0.9800	C18—H18	0.9500
C7—H7B	0.9800		
			
C1—O1—H1O	101 (3)	H9A—C9—H9B	109.5
C8—N1—N2	118.6 (2)	C8—C9—H9C	109.5
C10—N2—N1	121.3 (2)	H9A—C9—H9C	109.5
C10—N2—H2N	121 (3)	H9B—C9—H9C	109.5
N1—N2—H2N	118 (3)	O2—C10—N2	119.0 (3)
C13—N3—C19	109.0 (2)	O2—C10—C11	122.4 (3)
C13—N3—H3N	125 (2)	N2—C10—C11	118.6 (2)
C19—N3—H3N	126 (2)	C12—C11—C10	114.4 (2)
O1—C1—C2	117.0 (3)	C12—C11—H11A	108.7
O1—C1—C6	122.2 (2)	C10—C11—H11A	108.7
C2—C1—C6	120.8 (3)	C12—C11—H11B	108.7
C3—C2—C1	120.4 (3)	C10—C11—H11B	108.7
C3—C2—H2	119.8	H11A—C11—H11B	107.6
C1—C2—H2	119.8	C13—C12—C14	106.0 (3)
C2—C3—C4	121.0 (3)	C13—C12—C11	125.4 (3)
C2—C3—H3	119.5	C14—C12—C11	128.6 (3)
C4—C3—H3	119.5	N3—C13—C12	110.4 (3)
C5—C4—C3	117.7 (3)	N3—C13—H13	124.8
C5—C4—C7	120.6 (3)	C12—C13—H13	124.8
C3—C4—C7	121.8 (3)	C15—C14—C19	118.7 (3)
C4—C5—C6	123.4 (3)	C15—C14—C12	134.3 (3)
C4—C5—H5	118.3	C19—C14—C12	107.0 (3)
C6—C5—H5	118.3	C16—C15—C14	119.2 (3)
C5—C6—C1	116.8 (3)	C16—C15—H15	120.4
C5—C6—C8	121.1 (3)	C14—C15—H15	120.4
C1—C6—C8	122.1 (3)	C15—C16—C17	121.0 (3)
C4—C7—H7A	109.5	C15—C16—H16	119.5
C4—C7—H7B	109.5	C17—C16—H16	119.5
H7A—C7—H7B	109.5	C18—C17—C16	121.2 (3)
C4—C7—H7C	109.5	C18—C17—H17	119.4
H7A—C7—H7C	109.5	C16—C17—H17	119.4
H7B—C7—H7C	109.5	C17—C18—C19	117.5 (3)
N1—C8—C6	116.3 (3)	C17—C18—H18	121.3
N1—C8—C9	123.2 (3)	C19—C18—H18	121.3
C6—C8—C9	120.5 (2)	N3—C19—C18	129.9 (3)
C8—C9—H9A	109.5	N3—C19—C14	107.6 (2)
C8—C9—H9B	109.5	C18—C19—C14	122.4 (3)

### Hydrogen-bond geometry (Å, °)

**Table:**

*D*—H···*A*	*D*—H	H···*A*	*D*···*A*	*D*—H···*A*
O1—H1o···N1	0.85 (3)	1.75 (2)	2.540 (3)	153 (4)
N2—H2n···O2^i^	0.85 (3)	2.05 (3)	2.884 (3)	166 (4)
N3—H3n···O1^ii^	0.85 (3)	2.08 (3)	2.913 (3)	166 (3)

Symmetry codes: (i) −*x*+1, −*y*, −*z*+1; (ii) −*x*+1, −*y*+1, −*z*+1.
